# Gene Therapy in Movement Disorders: A Systematic Review of Ongoing and Completed Clinical Trials

**DOI:** 10.3389/fneur.2021.648532

**Published:** 2021-04-06

**Authors:** Aristide Merola, Noelle Kobayashi, Alberto Romagnolo, Brenton A. Wright, Carlo Alberto Artusi, Gabriele Imbalzano, Irene Litvan, Amber D. Van Laar, Krystof Bankiewicz

**Affiliations:** ^1^Department of Neurology, Madden Center for Parkinson Disease and Other Movement Disorders, The Ohio State University Wexner Medical Center, Columbus, OH, United States; ^2^The Ohio State University, Columbus, OH, United States; ^3^Department of Neuroscience “Rita Levi Montalcini,” University of Turin, Turin, Italy; ^4^Department of Neurosciences, Parkinson and Other Movement Disorders Center, University of California, San Diego, La Jolla, CA, United States; ^5^Asklepios BioPharmaceutical Inc., Research Triangle, NC, United States; ^6^Department of Neurology, University of Pittsburgh Medical Center, Pittsburgh, PA, United States; ^7^Department of Neurological Surgery, College of Medicine, The Ohio State University, Columbus, OH, United States

**Keywords:** movement disorder, multiple system atrophy, amino acid decarboxylase deficiency, ataxia, Huntington, Parkinson, gene therapy

## Abstract

**Introduction:** We sought to provide an overview of the published and currently ongoing movement disorders clinical trials employing gene therapy, defined as a technology aiming to modulate the expression of one or more genes to achieve a therapeutic benefit.

**Methods:** We systematically reviewed movement disorders gene therapy clinical trials from PubMed and ClinicalTrials.gov using a searching strategy that included Parkinson disease (PD), Huntington disease (HD), amino acid decarboxylase (AADC) deficiency, multiple system atrophy (MSA), progressive supranuclear palsy (PSP), dystonia, tremor, ataxia, and other movement disorders. Data extracted included study characteristics, investigational product, route of administration, safety/tolerability, motor endpoints, and secondary outcomes (i.e., neuroimaging, biomarkers).

**Results:** We identified a total of 46 studies focusing on PD (21 published and nine ongoing), HD (2 published and 5 ongoing), AADC deficiency (4 published and 2 ongoing), MSA (2 ongoing), and PSP (1 ongoing). In PD, intraparenchymal infusion of viral vector-mediated gene therapies demonstrated to be safe and showed promising preliminary data in trials aiming at restoring the synthesis of dopamine, enhancing the production of neurotrophic factors, or modifying the functional interaction between different nodes of the basal ganglia. In HD, monthly intrathecal delivery of an antisense oligonucleotide (ASO) targeting the huntingtin protein (HTT) mRNA proved to be safe and tolerable, and demonstrated a dose-dependent reduction of the cerebrospinal fluid levels of mutated HTT, while a small phase-I study testing implantable capsules of cells engineered to synthesize ciliary neurotrophic factor failed to show consistent drug delivery. In AADC deficiency, gene replacement studies demonstrated to be relatively safe in restoring catecholamine and serotonin synthesis, with promising outcomes. Ongoing movement disorders clinical trials are focusing on a variety of gene therapy approaches including alternative viral vector serotypes, novel recombinant genes, novel delivery techniques, and ASOs for the treatment of HD, MSA, and distinct subtypes of PD (LRRK2 mutation or GBA1 mutation carriers).

**Conclusion:** Initial phase-I and -II studies tested the safety and feasibility of gene therapy in PD, HD, and AADC deficiency. The ongoing generation of clinical trials aims to test the efficacy of these approaches and explore additional applications for gene therapy in movement disorders.

## Introduction

Gene therapy is a rapidly evolving technology aiming to modulate the expression of one or more genes to achieve a clinical therapeutic benefit (1). Several gene therapy studies have now been conducted in movement disorders and other areas of neurology to target the pathogenesis of diseases, enhance the production of key enzymes involved in neurotransmitter metabolic pathways, or support the survival of specific cell populations.

Gene therapy uses vectors to convey complementary DNA (cDNA), guide-RNA, small interfering RNAs (siRNA), microRNAs, or docking sites for DNA binding proteins (2). The ability to vary the type of genetic modulation lends a gene therapy approach to potentially be applied to a broad range of clinical indications within the central nervous system (CNS) and beyond. Genes encoding therapeutic proteins can be delivered via cDNA to replace a missing or mutated protein, modulate the concentration of a particular enzyme, or enrich the cellular microenvironment by enhancing the production of neurotrophic factors. In contrast, gene silencing strategies may treat diseases with an associated toxic gain of function mutation by using siRNAs or microRNAs to interfere with and degrade mRNA, decreasing protein translation and the resultant protein production (3). More innovative techniques involve the addition or alteration of polyadenylation signals, splicing of introns, and open reading frames (ORF) to modulate levels of gene expression (4), or the use of an RNA-guided Cas9 nuclease to target a specific region of the genome for targeted editing (5).

In this systematic review, we sought to report the state-of-the-art advancements in gene therapy related to movement disorders by critically reviewing and discussing the results of published studies and ongoing clinical trials.

## Methods

We conducted a systematic review in accordance with the Preferred Reporting Items for Systematic Reviews and Meta-analyses (PRISMA) guideline (6), including both published data and available data from ongoing gene therapy clinical trials for the treatment of movement disorders. For the purpose of this review, gene therapy was defined as a therapeutic intervention aimed at modulating the expression of a gene though the use of DNA or RNA encoding a protein (i.e., trophic factor, enzyme), antisense oligonucleotide interfering with RNA translation, or DNA-/RNA-editing enzymes (1). In addition, we included studies based on the use of cells engineered to produce specific proteins, such as neurotrophic factors.

### Search Methods

We searched for eligible studies in Pubmed and ClinicalTrials.gov, until October 2020 using the following search terms: “Parkinson Disease [MeSH Terms],” “Huntington Disease [MeSH Terms],” “Ataxia [MeSH Terms],” “Dystonia [MeSH Terms],” “Movement Disorders [MeSH Terms],” “genetic therapy [MeSH Terms],” “case report,” “AAV,” “adenovirus,” “adeno,” “lentivirus,” “ASO,” “antisense oligonucleotide,” “antisense” (Supplementary Table 1).

The “clinical trial” filter was applied for all searches except those including the term “case report.” Only studies referring to human subjects published in English were considered; no restrictions were applied to gender, age, ethnicity, follow-up duration, disease duration, or disease severity. The reference lists of retrieved articles were searched to obtain any other pertinent studies or search terms that were not captured by the original searching strategies.

### Selection of Studies and Data Extraction

Abstracts and pertinent full-text articles were independently reviewed for eligibility criteria by one author (NK) and double-checked by two additional authors (CA; AR). Duplicated studies were identified and excluded. A data collection form was used to extract variables of interest from the selected studies. Particular attention was paid to studies that shared the same population or published data from the same cohort at different time-points: in these cases, data from all the available manuscripts were reported, specifying how many participants were included in more than one trial. Disagreements were anticipated to be settled by consensus.

Data collected included year of publication; study design; sample size; sex, age, and disease duration at the time of intervention; dose, target, and route of administration of the compound delivered; follow-up duration; and key adverse events (AEs). In addition, the following data were acquired for each individual population.

#### Parkinson Disease (PD)

Safety and tolerability, with particular attention to AE reporting; motor section of the Unified Parkinson's Disease Rating Scale (UPDRS-III); Levodopa Equivalent Daily Dose (LEDD) both before intervention and at follow-up. Depending on the endpoints evaluated and the outcome measures used in each trial, we reported changes in motor fluctuation scales (i.e., UPDRS-IV, or time of the waking day spent in Off-state, or in On-state without troublesome dyskinesia), in activities of daily living (ADL) scales [i.e., UPDRS-II, Schwab and England (S&E) scale], in quality of life (QoL) scales (i.e., Parkinson's disease Questionnaire 39—PDQ-39), in cognition scales (i.e., UPDRS-I, neuropsychological batteries), and changes in regional metabolic activity (i.e., PET ligand uptake). Finally, for post-mortem studies, we evaluated the enzymatic expression or immunoreactivity and the area of the targeted brain region covered by the compound delivered.

#### Huntington Disease (HD)

Safety and tolerability, with particular attention to AE reporting; changes in the Unified Huntington's Disease Rating Scale (UHDRS) and in the Total Functional Capacity Scale (TFC). Depending on the endpoints evaluated and the outcome measures used in each trial, we reported changes in functional, cognitive, and psychiatric outcomes, changes in regional metabolic activity (i.e., PET ligand uptake), and in pharmacokinetics variables.

#### Aromatic L-Amino Acid Decarboxylase (AADC) Deficiency

Safety and tolerability, with particular attention to AE reporting, and the following secondary endpoints: changes in motor, cognitive, and autonomic functions.

Data were expressed as a mean with standard deviation (SD) or proportion, as appropriate.

### Assessment of Risk of Bias

Two investigators (NK; GI) independently assessed the quality appraisal of pertinent studies. A third investigator (C.A.A.) checked the quality assessment and any disagreement in the scoring was resolved by discussion between the three examiners. Given the heterogeneity of study designs, the risk of bias of individual studies was appraised utilizing the National Heart, Lung, and Blood Institute tools (NHLBI, Research Triangle Institute International. National Heart, Lung, and Blood Institute Quality Appraisal Tools), following the Cochrane handbook recommendations (7). Pooled studies were assessed to determine the overall quality of evidence (Tables 1, 2).

**Table 1:** Reviewed articles—baseline features—Parkinson disease.

**Study**	**Quality rating**	**No. of patients (Active/Sham)**	**Gender (M/F)**	**Age (years)**	**Disease duration (years)**	**UPDRS-III at baseline**	**LEDD at baseline (mg)**
**AAV2-AADC/BILATERAL PUTAMEN**							
Eberling et al. (8)^*^	Fair	5/0	1/4	63.0 ± 3.1	10.8 ± 7.5	40.5 ± 1.6 (Off-state)	910.6 ± 419.8
Open-label/Phase I						15.8 ± 1.9 (On-state)	
Christine et al. (9)^*^	Fair	10/0	5/5	62.7 ± 4.1	9.3 ± 5.3	38.6 ± NR (Off-state)	1,049.9 ± 395.9
Open-label/Phase I						15.5 ± NR (On-state)	
Valles et al. (10)^*^	Poor	10/0	5/5	62.7 ± 4.1	9.3 ± 5.3	38.6 ± NR (Off-state)	1,049.9 ± 395.9
Open-label/Phase I						15.5 ± NR (On-state)	
Muramatsu et al. (11)	Fair	6/0	4/2	60.0 ± 6.5	10.0 ± 4.5	25.3 ± 9.4 (Off-state)	808.0 ± 169.0
Open-label/Phase I						5.2 ± 4.6 (On-state)	
Mittermeyer et al. (12)^*^	Fair	10/0	5/5	62.7 ± 4.1	9.3 ± 5.3	38.6 ± NR (Off-state)	1,049.9 ± 395.9
Open-label/Phase I						15.5 ± NR (On-state)	
Christine et al. (13)	Good	15/0	13/2	57.7 ± 1.7	9.5 ± 0.9	37.1 ± 1.9 (Off-state)	1,526.5 ± 141.9
Open-label/Phase I						13.5 ± 1.5 (On-state)	
**AAV2-GAD/UNILATERAL STN**							
Kaplitt et al. (14)	Good	12/0	11/1	58.2 ± 5.7	9.3 ± 2.7	39.2 ± 7.5 (Off-state)	1,401.8 ± 723.6
Open-label/Phase I						22.1 ± 8 (On-state)	
**AAV2-GAD/BILATERAL STN**							
Lewitt et al. (15)^*^	Good	16/21	12/4 (active)	61.8 ± 7.0 (active)	10.6 ± 4.3 (active)	34.8 ± 6.6 (active—Off-state)	1,149.0 ± 536.3 (active)
Double-blind RCT/Phase II			15/6 (sham)	60.6 ± 7.4 (sham)	12.0 ± 5.0 (sham)	39.0 ± 8.7 (sham—Off-state)	1,125.9 ± 493.8 (sham)
						18.7 ± 2.2 (active—On-state)	
						20.0 ± 2.0 (sham—On-state)	
Niethammer et al. (16)^*^	Good	16/21	12/4 (active)	61.8 ± 7.0 (active)	10.6 ± 4.3 (active)	34.8 ± 6.6 (active—Off-state)	1,149.0 ± 536.3 (active)
Open-label/Phase II			15/6 (sham)	60.6 ± 7.4 (sham)	12.0 ± 5.0 (sham)	39.0 ± 8.7 (sham—Off-state)	1,125.9 ± 493.8 (sham)
						18.7 ± 2.2 (active—On-state)	
						20.0 ± 2.0 (sham—On-state)	
Niethammer et al. (17)^*^	Fair	16/21	12/4 (active)	61.8 ± 7.0 (active)	10.6 ± 4.3 (active)	34.8 ± 6.6 (active—Off-state)	1,149.0 ± 536.3 (active)
Open-label/Phase II			15/6 (sham)	60.6 ± 7.4 (sham)	12.0 ± 5.0 (sham)	39.0 ± 8.7 (sham—Off-state)	1,125.9 ± 493.8 (sham)
						18.7 ± 2.2 (active—On-state)	
						20.0 ± 2.0 (sham—On-state)	
**LV-TH/CH1/AADC/BILATERAL PUTAMEN**							
Palfi et al. (18)^*^	Good	15/0	NR	57.4 ± 4.3	13.9 ± 5.3	38.4 ± 9.3 (Off-state)	1,687.2 ± 487.7
Open-label/Phase I/II						12.8 ± 5.5 (On-state)	
Palfi et al. (19)^*^	Fair	15/0	NR	57.4 ± 4.3	13.9 ± 5.3	38.4 ± 9.3 (Off-state)	1,687.2 ± 487.7
Open-label/Phase I/II						12.8 ± 5.5 (On-state)	
**AAV2-GDNF/BLATERAL PUTAMEN**							
Heiss et al. (20)	Fair	13/0	10/3	61.6 ± 6.5	12.9	38.5 ± 12.9 (Off-state)	1,282.2 ± 325.4
Open-label/Phase I						27.5 ± 13.5 (On-state)	
**AAV2-NRTN/BILATERAL PUTAMEN**							
Marks et al. (21)	Good	12/0^*^	9/3	57.0 ± 8.0	11.0 ± 3.2	42.0 ± 9.5 (Off-state)	1,783 ± 1,651.6
Open-label/Phase I						13.0 ± 7.7 (On-state)	
Marks et al. (22)	Good	38/20	28/10 (active)	60.1 ± 7.6 (active)	9.5 ± 3.4 (active)	38.7 ± 8.5 (active—Off-state)	1,046.9 ± 551.8 (active)
Double-blind RCT/Phase IIa			15/5 (sham)	57.3 ± 8.3 (sham)	10.0 ± 4.9 (sham)	17.1 ± 7.5 (active—On-state)	1,108.7 ± 640.7 (sham)
						39.0 ± 9.4 (sham—Off-state)	
						16.6 ± 7.6 (sham—On-state)	
**AAV2-NRTN/BILATERAL PUTAMEN AND SN**							
Bartus et al. (23)	Fair	6/0	4/2	50.3 ± 7.4	8.8 ± 3.7	38.2 ± 6.4 (Off-state)	NR
Open-label/Phase I						10.2 ± 4.3 (On-state)	
Olanow et al. (24)	Good	23/25	15/8 (active)	59.7 ± 5.5 (active)	7.8 ± 3.2 (active)	35.2 ± 6.9 (active—Off-state)	1,163.3 ± 447.9 (active)
Double-blind RCT/Phase IIb			17/8 (sham)	58.7 ± 7.1 (sham)	8.6 ± 3.3 (sham)	15.7 ± 5.8 (active—On-state)	1,208.3 ± 514.5 (sham)
						36.4 ± 14.2 (sham—Off-state)	
						12.2 ± 7.3 (sham—On-state)	
Marks et al. (25)	Fair	53/0	See data from (21–23)	See data from (21–23)	See data from (21–23)	See data from (21–23)	See data from (21–23)
Long-term FUP of Phase I and II studies							
**POST-MORTEM STUDIES**							
Bartus et al. (26)^*^	Fair	2/0	2/0	59 and 73	10.2 and 9.5	34/21 and 51/34 (Off/On)	NR
Bartus et al. (27)^**^	Fair	4/0	4/0	59–74	5–10.2	NR	NR
Chu et al. (28)^***^	Fair	2/0	NR	75 and 50	14 and 12	38/16.5 and 35.5/16 (Off/On)	NR

*AADC, aminoacid decarboxylase; AAV2, Adeno-Associated Viral Vector 2; GAD, Glutamic Acid Decarboxylase Gene; GDNF, Glial cell line-Derived Neurotrophic Factor; LEDD, Levodopa Equivalent Daily Dose; LV, Lentivirus; NR, Not Reported; NRTN, Neurturin; pts, patients; SN, Substantia Nigra; STN, Subthalamic Nucleus; UPDRS, Unified Parkinson's Disease Rating Scale*.

AADC Studies: *Four different studies from the same trial, with different endpoints or follow-up.

GAD Studies: *Three different studies from the same trial, with different endpoints or follow-up.

LV-TH/CH1/AADC Studies: *Two different studies from the same cohort, with different endopoints or follow-up.

NRTN Post-mortem studies:

* 2 patients from Marks et al. (22);

** 3 patients from Marks et al. (22) [of which two already described in (26)] and 1 patient from Marks et al. (21);

*** *1 patient from Marks et al. (22) and 1 patient from Bartus et al. (23)*.

**Table 2:** Reviewed articles—baseline features—other diseases.

**Study**	**Quality rating**	**No. of patients (Active/Sham)**	**Gender (M/F)**	**Age (years)**	**Disease duration (years)**	**Therapy at baseline**
**HUNTINGTON DISEASE**—**CNTF**/**RIGHT VENTRICLE IMPLANTATION**						
Bloch et al. (29)	Fair	6/0	3/3	46.3 ± 4.8	6.8 ± 4.7	NR
Open-label/Phase I						
**HUNTINGTON DISEASE**—**ASO-HTT**_**Rx**_/**INTRATHECAL INFUSION**						
Tabrizi et al. (30)	Good	34/12	20/14 (active)	46.0 ± 10.0 (active)	NR	NR
Double-blind RCT/Phase I/IIa			8/4 (sham)	49.0 ± 10.0 (sham)		
**AADC DEFICIENCY**—**AAV2-AADC**/**BILATERAL PUTAMEN**						
Hwu et al. (31)	Fair	4/0	1/3	4.7± 0.9	4–6	NR
Open-label/Phase I						
Chien et al. (32)	Fair	10/0	5/5	4.3 ± 2.4	3.2 ± 2.6	NR
Open-label/Phase I/II						
Kojima et al. (33) Open-label/Phase I/II	Fair	6/0	4/2	10.8 ± 5.8	10.8 ± 5.8	Vitamin B6 (5 pts), L-DOPA (2 pts), dopamine agonist (3 pts), MAOB inhibitor (3 pts), SSRI (2 pts)
**AADC DEFICIENCY**—**AAV2-AADC**/**BILATERAL SN AND VTA**						
Pearson et al. (34)	Fair	7/0	4/3	6.5 ± 1.6	6.5 ± 1.6	NR
Open-label/Phase I						

*AADC, aminoacid decarboxylase; AAV2, Adeno-Associated Viral Vector 2; CNTF, Ciliary Neurotrophic Factor; HTTRx, Antisense Oligonucleotide Targeting HTT mRNA; NR, Not Reported; pts, patients; SN, Substantia Nigra; SSRI, selective serotonin reuptake inhibitor; VTA, Ventral Tegmental Area*.

## Results

**Figure 1:**
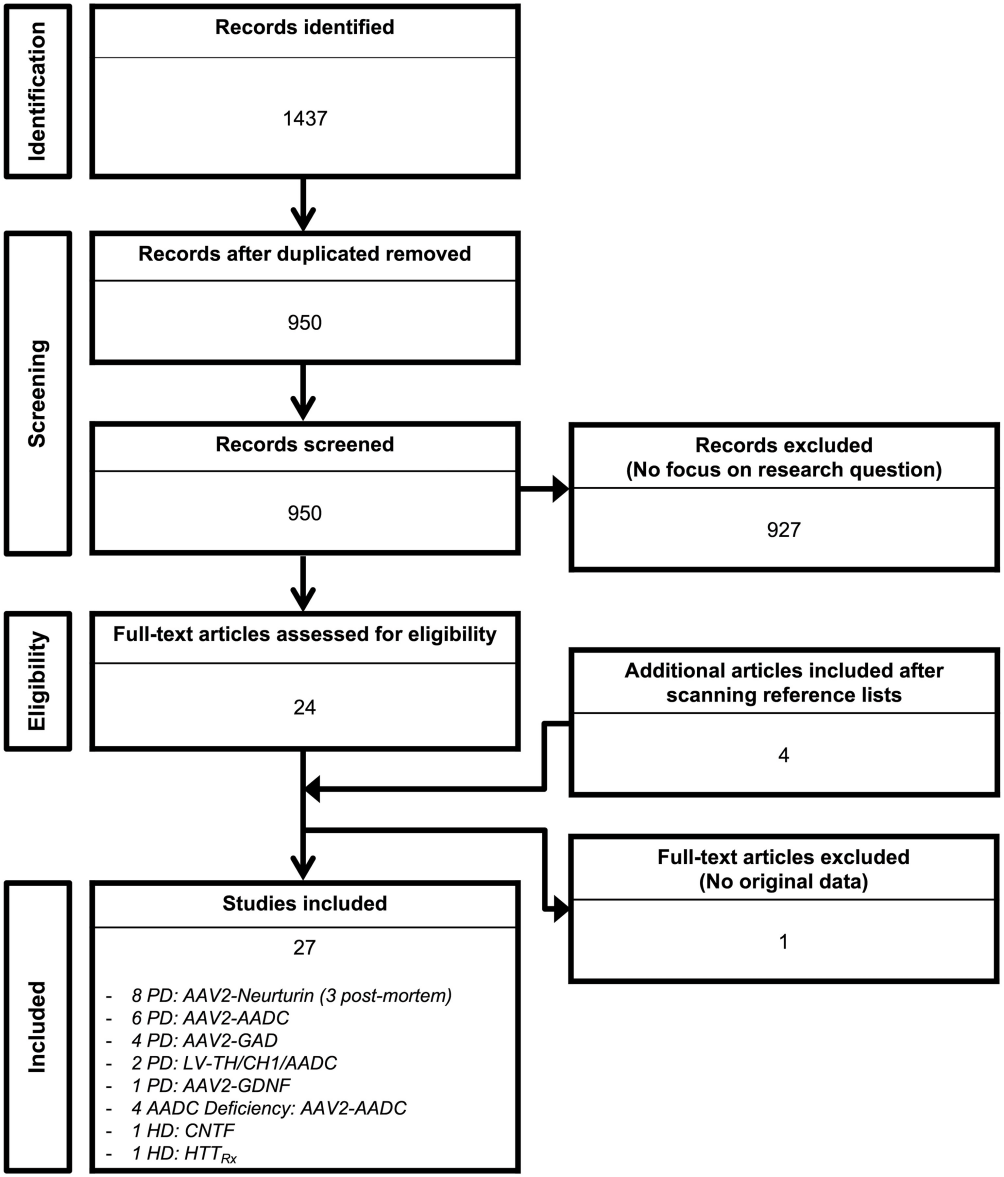
Search flow diagram. Graphical representation of the studies identified, screened for eligibility, and included in the review.

Out of the 1,437 studies derived from the initial searching strategy (Figure 1), 27 published articles met full criteria (8–34) (Tables 1–**6**) and underwent data extraction and assessment for individual risk of bias. There were 21 studies focusing on PD (8–28), two on HD (29, 30), and four on AADC deficiency (31–34).

The searching strategy identified eight distinct gene therapy-based molecular approaches: AADC (*n* = 10 studies) (8–13, 31–34); Ciliary Neurotrophic Factor (CNTF; *n* = 1 study) (29); Glutamic acid decarboxylase (GAD; *n* = 4 studies) (14–17); Glial cell line-derived neurotrophic factor (GDNF; *n* = 1 study) (20); antisense oligonucleotide targeting HTT mRNA (HTT_Rx_; *n* = 1 study) (30); Neurturin (NRTN; *n* = 8 studies) (21–28); and Prosavin (*n* = 2 studies), a multigene approach including tyrosine hydroxylase (TH), GTP-cyclohydrolase 1 (CH1), and AADC (18, 19).

There were 25 studies investigating intraparenchymal gene therapy delivery using Adeno-Associated Virus type 2 (AAV2; *n* = 19 PD studies, and *n* = 4 AADC deficiency study) (8–17, 20–28, 31–34) or lentivirus (LV; *n* = 2 PD studies) (18, 19), 1 study of direct ventricular implantation of polymer-encapsulated cells in HD (29), 1 study of ASO intrathecal infusion in HD (30).

### Parkinson Disease

A total of 166 participants with PD received active treatment, and 66 underwent a sham procedure as part of the following studies: AAV2-AADC (*n* = 3 studies) (8–13), AAV2-GAD (*n* = 4 studies) (14–17), ProSavin (*n* = 2 studies) (18, 19), AAV2-GDNF (*n* = 1 study) (20), and AAV2-NRTN (*n* = 8 studies, three of which reporting post-mortem data) (21–28) (Tables 1, 3, 4). In all cases, the intraparenchymal delivery was performed via stereotactic neurosurgery.

**Table 3:** Reviewed articles—sample characteristics and endpoints—Parkinson disease.

**Study**	**Dose and volume delivered**	**Follow-up (months)**	**Primary endpoint**	**Other endpoints**
**AAV2-AADC**/**BILATERAL PUTAMEN**				
Eberling et al. (8)^*^	9 × 10^10^ vg (5 pts)−200 μL	6	Changes in putaminal FMT-PET	Adverse events and clinical efficacy
Open-label/Phase I			uptake (AADC tracer)	
Christine et al. (9)^*^	9 × 10^10^ vg/mL (5 pts)−200 μL	6	Safety and tolerability	Motor symptoms, fluctuations, and LEDD
Open-label/Phase I	3 × 10^11^ vg/mL (5 pts)−200 μL			Changes in putaminal FMT-PET uptake
Valles et al. (10)^*^	9 × 10^10^ vg (5 pts)−200 μL	6	Putamen targeting accuracy (MRI)	Volume of vector distribution (MRI) and
Open-label/Phase I	3 × 10^11^ vg (5 pts)−200 μL			correlation with FMT-PET uptake
Muramatsu et al. (11)	3 × 10^11^ vg (6 pts)−200 μL	6	Safety and tolerability	Motor symptoms, fluctuations, and LEDD
Open-label/Phase I				Changes in putaminal FMT-PET uptake
Mittermeyer et al. (12)^*^	9 × 10^10^ vg (5 pts)−200 μL	09	Long-term gene expression	Safety and tolerability
Open-label/Phase I	3 × 10^11^ vg (5 pts)−200 μL		(FMT-PET)	Effect on total UPDRS
Christine et al. (13)	7.5 × 10^11^ vg (5 pts)−450 μL	18–36	Safety and tolerability of the	AADC activity changes in response to
Open-label/Phase I	1.5 × 10^12^ vg (5 pts)−900 μL		MRI-guided administration technique	levodopa
	4.7 × 10^12^ vg (5 pts)−900 μL			Clinical outcomes over 12 months Stability of change after 1 year
**AAV2-GAD**/**UNILATERAL STN**				
Kaplitt et al. (14)	1 × 10^11^ vg/mL. (4 pts)−50 μL	12	Safety and tolerability	Motor symptoms, ADLs, cognition, and
Open-label/Phase I	3 × 10^11^ vg/mL (4 pts)−50 μL			LEDD
	1 × 10^12^ vg/mL (4 pts)−50 μL			Changes in regional metabolic activity (FDG-PET)
**AAV2-GAD**/**BILATERAL STN**				
Lewitt et al. (15)^*^	1 × 10^12^ vg/mL−69 μL	6	Motor symptoms	Motor fluctuations, quality of life, and
Double-blind RCT/Phase II				cognition
Niethammer et al. (16)^*^	1 × 10^12^ vg/mL−69 μL	12	Motor symptoms	Motor fluctuations, ADLs, and cognition
Open-label/Phase II				Regional metabolic activity (FDG-PET)
Niethammer et al. (17)^*^	1 × 10^12^ vg/mL−69 μL	12	Metabolic network analysis	NA
Open-label/Phase II				
**LV-TH/CH1/AADC**/**BILATERAL PUTAMEN**				
Palfi et al. (18)^*^ Open-label/Phase I/II	1.9 × 107 TU (3 pts); 4 × 107 TU (6 pts); 1 × 108 TU (6 pts)	12	Safety and motor symptoms	Motor fluctuation, QoL, LEDD, and cognition Changes in putaminal 18F-Ldopa uptake
	- Volume not recorded			
Palfi et al. (19)^*^ Open-label/Phase I/II	1.9 × 107 TU (3 pts); 4 × 107 TU (6 pts); 1 × 108 TU (6 pts)	24–72	Safety and motor symptoms	Motor fluctuations, QoL, and LEDD
	- Volume not recorded			
**AAV2-GDNF**/**BILATERAL PUTAMEN**				
Heiss et al. (20)	9 × 10^10^ vg (6 pts)−900 uL	18	Safety and tolerability	Effect on total UPDRS and LEDD
Open-label/Phase I	3 × 10^11^ vg (6 pts)−900 uL			Changes in putaminal 18F-Ldopa uptake
	9 × 10^11^ vg (1 pt)−900 uL			
**AAV2-NRTN**/**BILATERAL PUTAMEN**				
Marks et al. (21)	1.3 × 10^11^ vg (6 pts)−80 μL	12	Safety and tolerability	Motor symptoms, motor fluctuation, and
Open-label/Phase I	5.4 × 10^11^ vg (6 pts)−80 μL			cognition
				Changes in putaminal 18F-Ldopa uptake
Marks et al. (22) Double-blind RCT/Phase IIa	5.4 × 10^11^ vg−80 μL	12–18	Motor symptoms	Motor fluctuation, ADLs, and QoL Safety
**AAV2-NRTN**/**BILATERAL PUTAMEN AND SN**				
Bartus et al. (23) Open-label/Phase I	4.0 × 10^11^ vg in SN and 5.4 × 10^11^ vg in putamen (3 pts)	24	Safety and tolerability	Motor symptoms, motor fluctuations, and QoL
	- Volume not recorded			
	4.0 × 10^11^ vg in SN and 24.0 × 10^11^ vg in putamen (3 pts)			
	- Volume of 360 μL in the putamen			
Olanow et al. (24) Double-blind RCT/Phase IIb	4.0 × 10^11^ vg in SN-30 μL and 2.0 × 10^12^ vg in putamen-150 μL	15–24	Motor symptoms in Off-state	Motor fluctuations, ADL, QoL, and non-motor symptoms
				Safety
Marks et al. (25)	See data from (21–23)	60	Safety	Motor symptoms and motor fluctuation
Long-term FUP of Phase I and II studies				
**POST-MORTEM STUDIES**				
Bartus et al. (26)^*^	5.4 × 10^11^ vg−80 μL	1.5 and 3	Target covered area, NRTN and TH expression	NA
Bartus et al. (27)^**^	1.3 × 10^11^ vg (1 pt)−80 μL 5.4 × 10^11^ vg (3 pts)−80 μL	1.5; 3; 48; 52	Target covered area, NRTN and TH expression	NA
Chu et al. (28)^***^	5.4 × 10^11^ vg in putamen (1 pt)−80 μL	96 and 120	Target covered area, NRTN TH, and RET/TH expression	Persistence of vg
	4.0 × 10^11^ vg in SN and 24.0 × 10^11^ vg in putamen (1 pt)−360 μL			

*AADC, aminoacid decarboxylase; AAV2, Adeno-Associated Viral Vector 2; FDG-PET, 18F-fluorodeoxyglucose PET scan; FMT-PET, 18F-fluoro-L-m-tyrosine PET scan; GAD, Glutamic Acid Decarboxylase Gene; GDNF, Glial cell line-Derived Neurotrophic Factor; LEDD, Levodopa Equivalent Daily Dose; LV, Lentivirus; MRI, Magnetic Resonance Imaging; NA, Not Applicable; NR, Not Reported; NRTN, Neurturin; PDQ-39, Parkinson's disease Questionnaire 39; pts, patients; RET, RET proto-oncogene; SN, Substantia Nigra; STN, Subthalamic Nucleus; TH, tyrosine Hydroxylase; TU, Transducing units; UPDRS, Unified Parkinson's Disease Rating Scale; vg, vector genomes*.

AADC Studies: *Four different studies from the same trial, with different endpoints or follow-up.

GAD Studies: *Three different studies from the same trial, with different endpoints or follow-up.

LV-TH/CH1/AADC Studies: *Two different studies from the same cohort, with different endopoints or follow-up.

NRTN Post-mortem studies:

* 2 patients from Marks et al. (22);

** 3 patients from Marks et al. (22) [of which two already described in (26)] and one patient from Marks et al. (21);

*** *1 patient from Marks et al. (22) and 1 patient from Bartus et al. (23)*.

**Table 4:** Reviewed articles—efficacy and safety—Parkinson disease.

**Study**	**Efficacy**	**Adverse events**
**AAV2-AADC**/**BILATERAL PUTAMEN**		
Eberling et al. (8)^*^ Open-label/Phase I	−28% total UPDRS and 34% UPDRS-III improvement (Off- state) - 33% total UPDRS improvement (On-state) - No significant changes in UPDRS-III (On-state), On time, or LEDD	- One asymptomatic intracranial hemorrhage related to surgery [same of (9)] - Self-limited headache and surgical site pain
	- 30% increase in FMT uptake	
Christine et al. (9)^*^ Open-label/Phase I	- 31% UPDRS and 36% UPDRS-III improvement (Off-state). - 32% total UPDRS improvement (On-state) - 3.1 h improvement in Off-time - No significant changes in UPDRS-III (On-state), On-time, LEDD	- Three intracranial hemorrhages related to surgery (two asymptomatic, one with transient hemiplegia and aphasia with almost complete recovery)
	- 30% (low-dose) and 75% (high dose) increase in FMT uptake	
Valles et al. (10)^*^ Open-label/Phase I	-Surgical accuracy confirmed by MRI (halo of T2 hyperintensity around cannula tip)	NR
	- 30% (low-dose) and 75% (high dose) increase in FMT uptake [same of (9)]	
	- MRI sites of hyperintensity coregistered with areas of increased PET uptake.	
Muramatsu et al. (11) Open-label/Phase I	- 28% total UPDRS and 45% UPDRS-III improvement in (Off-state) - No significant changes in On-state UPDRS scores, On time, or LEDD	- One venous hemorrhage (with transient left arm weakness and complete recovery)
	- 56% increase in FMT uptake	
Mittermeyer et al. (12)^*^ Open-label/Phase I	- UPDRS improvement (Off- and On-state) after 12 months, followed by a return to pre-surgery values	- Three intracranial hemorrhage related to surgery [same as (9)] - Four patients with infusion-related increase in dyskinesia
	- 25% (low-dose) and 65% (high dose) increase in FMT uptake	
Christine et al. (13) Open-label/Phase I	- UPDRS-III (Off-state) and On-time without dyskinesia improvement in all dose cohorts - LEDD decrease in all cohorts - UPDRS-III (On-state) improvement in the two highest dose groups	- Four transient increase in dyskinesia - One patient with deep venous thrombosis, pulmonary embolus, and atrial fibrillation
**AAV2-GAD**/**UNILATERAL STN**		
Kaplitt et al. (14) Open-label/Phase I	- 24% UPDRS-III improvement (Off-state) - Reduction in thalamic metabolic activity in the operated hemisphere	-One severe freezing episode, one COPD exacerbation, one arthroscopic knee procedure (all unrelated to treatment)
**AAV2-GAD**/**BILATERAL STN**		
Lewitt et al. (15)^*^ Double-blind RCT/Phase II	- 8.1 points UPDRS-III (Off-state) improvement (vs. 4.7 in sham) - 0.5 points BPRS improvement (vs. 0 in sham) - No significant improvement in On-Off fluctuations or cognition	- One severe AE (bowel obstruction) unrelated to treatment - Only mild or moderate AEs probably or possibly related to treatment (headache and nausea)
Niethammer et al. (16)^*^	- 8.2 points UPDRS-III (Off-state) improvement (vs. 5 in sham)	Same of Lewitt et al. (15)
Open-label/Phase II	- Reduced dyskinesia duration	
	- No significant change in Off-time or cognition	
	- Decreased metabolic activity in the thalamus, striatum, prefrontal, anterior cingulate, and orbitofrontal cortices	
Niethammer et al. (17)^*^ Open-label/Phase II	- New polysynaptic functional pathways linking the STN to motor cortical regions	NR
**LV-TH/CH1/AADC**/**BILATERAL PUTAMEN**		
Palfi et al. (18)^*^	- 11.8 points UPDRS-III (Off-state) and 2 points UPDRS-IV improvement	- No serious drug-related AEs
Open-label/Phase I/II	- No significant change in LEDD, cognition, or 18F-Ldopa uptake	- 54 mild/moderate moderate drug-related AEs (dyskinesia, tremor, anxiety, on-off phenomena)
Palfi et al. (19)^*^ Open-label/Phase I/II	- 8.2 points UPDRS-III (Off-state) improvement - No significant improvements in UPDRS-I, -II, -III (On-state), -IV, PDQ-39, LEDD	Three severe drug-related AEs (dyskinesia, acute psychosis, nervous system disorder) and 93 mild/moderate AEs Two deaths at 4 and 6 years unrelated to treatment
**AAV2-GDNF**/**BILATERAL PUTAMEN**		
Heiss et al. (20) Open-label/Phase I	- Increase in 18F-Ldopa uptake - No significant changes in UPDRS scores or LEDD	-Two surgery-related AEs: one scalp wound dehiscence and one radiation exposure
		- Four severe AEs (two falls, one hemoptysis, one non-cardiac chest pain) and 423 non-severe AEs unrelated to treatment
**AAV2-NRTN**/**BILATERAL PUTAMEN**		
Marks et al. (21) Open-label/Phase I	- 36% UPDRS-III (Off-state), 18% UPDRS-IV, and 29% total UPDRS improvement	- No serious drug-related AEs - Mild, transient AEs related to surgery
	- 2.3 h/day increase in On-time without dyskinesia	
	- No significant change in UPDRS-III (On-state), cognition, or 18F-Ldopa uptake.	
Marks et al. (22)	- 33% UPDRS-III (Off-state) improvement (vs. 11% in sham)	- Two deaths in active group, unrelated to treatment
Double-blind RCT/Phase IIa	- Higher UPDRS-I and -II, On-time without dyskinesia, timed walking, CGI and PDQ-39 improvement in active vs. sham	- Three tumors in active group and two in sham group, not clearly related to treatment
**AAV2-NRTN**/**BILATERAL PUTAMEN AND SN**		
Bartus et al. (23) Open-label/Phase I	- 5.0 and 5.5 points UPDRS-III improvement (Off-state) at 12 and 24 months.	- No serious AEs - Mild, transient AEs related to surgery
	- 1.1 and 2.3 h decrease in Off-time at 12 and 24 months	
	- Stable PDQ-39 scores	
Olanow et al. (24) Double-blind RCT/Phase IIb	- No significant differences in UPDRS-III, ADL, QoL, or non-motor symptoms - Higher UPDRS-IV and Off-time improvement in active vs. sham (15-month *post-hoc* analysis)	- Two intracranial hemorrhages (mild symptoms), two coronary artery disease, and one ischemic stroke in active group, all unrelated to treatment
Marks et al. (25) Long-term FUP of Phase I and II studies	- UPDRS-III (Off-state) changes: from 3.2% worsening to 14.9% improvement - 1.9–3 h decrease in Off-time - 1.5–2.4 h increase in On-time	- Nine AEs possibly related to treatment: psychosis (3), dyskinesia (3), dizziness, insomnia, altered mood
**POST-MORTEM STUDIES**		
Bartus et al. (26)^*^	Putamen covered area: 15%; Detectable NRTN protein: 58–93%; Very little NRTN staining in the SN; TH-induction in 50% (0–80%) of NRTN-positive sites in putamen; No TH-induction in SN	
Bartus et al. (27)^**^	Putamen covered area: 8.5%; NRTN-positive neurons in SN: 0.3% (short-term) and 4.5% (long-term); TH immunoreactivity: 2.2% (short-term) and 13.4% (long-term) of total putaminal area; In all patients, numerous NRTN-laden neurons were seen in frontal cerebral cortex (layer V)	
Chu et al. (28)^***^	**Patient with putaminal delivery only**: Putamen covered area: 8.9–12.4%; SN covered area: 0%; TH immunoreactive density similar to non-PD control in putamen, similar to PD control in SN; RET/TH expression in nigral neurons lower than PD control;	
	**Patient with putaminal and SN delivery**: Putamen covered area: 3.7–4.0%; SN covered area: 56.9–66.6%; 2–3 fold higher density of TH+ fibers vs. patient with putaminal delivery only; TH immunoreactive density similar to non-PD control in putamen, and 1.7–3.3-fold higher than PD control in SN; RET/TH expression in nigral neurons higher than PD control; Low level of vg expression in both patients	

*AADC, aminoacid decarboxylase; AAV2, Adeno-Associated Viral Vector 2; AE, Adverse Event; BPRS, Brief Parkinsonism Rating scale; CGI, Clinical Global Impression; FMT-PET, 18F-fluoro-L-m-tyrosine PET scan; GAD, Glutamic Acid Decarboxylase Gene; GDNF, Glial cell line-Derived Neurotrophic Factor; LEDD, Levodopa Equivalent Daily Dose; LV, Lentivirus; MRI, Magnetic Resonance Imaging; NR, Not Reported; NRTN, Neurturin; PDQ-39, Parkinson's disease Questionnaire 39; RET, RET proto-oncogene; SN, Substantia Nigra; STN, Subthalamic Nucleus; TH, tyrosine Hydroxylase; TU, Transducing units; UPDRS, Unified Parkinson's Disease Rating Scale; vg, vector genomes*.

AADC Studies: *Four different studies from the same trial, with different endpoints or follow-up.

GAD Studies: *Three different studies from the same trial, with different endpoints or follow-up.

LV-TH/CH1/AADC Studies: *Two different studies from the same cohort, with different endopoints or follow-up.

NRTN Post-mortem studies:

* 2 patients from Marks et al. (22);

** 3 patients from Marks et al. (22) [of which two already described in (26)] and 1 patient from Marks et al. (21);

*** *1 patient from Marks et al. (22) and 1 patient from Bartus et al. (23)*.

#### AAV2-AADC

A total of 31 participants (22 males and 9 females) received AAV2-AADC through intraputaminal bilateral infusions over 6 phase-I open-label studies (8–13) (Tables 1, 3, 4). Four studies (8–10, 12) reported data on the same cohort of participants.

##### Motor Outcomes

One study (9) found a 36% Off-state UPDRS-III improvement at 6 months, followed by a gradual return to the pre-surgical baseline values after 48 months of follow-up (12) (Table 4). A similar extent of Off-state UPDRS-III improvement (range of 21–46% improvement from baseline) was observed in two additional studies (11, 13), after 6 and 18–39 months, respectively, with a trend toward greater improvement in those treated with higher AAV2-AADC dosages (Table 4). No significant changes were observed in the On-state UPDRS-III scores. Three studies reported an improvement in Off-time of 0.6–3.1 h per day (9, 11, 13) and one study (13) showed an improvement in the On-time without dyskinesia of 1.5–3.3 h per day.

##### Other Efficacy Outcomes

Four studies reported data on Fluoro-m-tyrosine PET (FMT-PET) analysis (9–12) showing a 25–75% increase in the putaminal uptake, with a trend toward greater increases in those treated with higher dosages (Table 4). One study reported 18F-DOPA PET, which demonstrated a 13–79% increase in enzyme activity, with a trend toward greater increases in the higher dose cohorts (13) (Table 4).

##### Safety

There were five serious AEs related to surgery: three intracranial hemorrhages (two asymptomatic) (8, 9, 12), one venous hemorrhage (11), and one deep venous thrombosis and subsequent pulmonary embolus (13) (Table 4). A transient increase in dyskinesia was also reported in eight participants (12, 13).

#### AAV2-GAD

A total of 28 participants (23 males and 5 females) received AAV2-GAD through subthalamic bilateral (*n* = 16) or unilateral infusion (*n* = 12) within the context of 1 phase I open-label study (14), 1 phase II double-blinded, randomized controlled trial (RCT) (15), and 2 phase II open-label studies (16, 17) (Tables 1, 3, 4). Three studies (15–17) reported data on the same cohort of participants.

##### AAV2-GAD (Bilateral Subthalamic Infusion)

*Motor outcomes.* Two phase II trials reported greater improvement of Off-state UPDRS-III scores in the active group compared to the sham surgery group at 6 months (8.1 vs. 4.7 points) (15) and 12 months (8.2 vs. 5 points) (16), but no significant differences were observed in the On-state UPDRS-III scores between groups (Table 4). Neither of the studies were able to demonstrate significant improvement of motor fluctuations, based on participants' diary or total UPDRS-IV. However, one study (16) found a significantly greater improvement of the UPDRS-IV item 32 (duration of dyskinesia) in the active vs. sham group (Table 4).

*Other efficacy outcomes.* Two studies reported data on functional imaging changes following treatment, showing significant ^18^F-fluorodeoxyglucose (FDG) PET activity decline in several brain regions (16) and the formation of new pathways linking the STN to other cortical motor regions (17) (Table 4). There were no significant changes in the PDQ-39, UPDRS-II, or any of the neuropsychiatric measures evaluated in either phase-II study. However, one study (15) reported a significant improvement in the Brief Parkinsonism Rating scale (Table 4).

*Safety data.* One serious adverse event (bowel obstruction) was reported in the AAV2-GAD group (15) and classified as unrelated to treatment. All other AEs possibly related to AAV2-GAD or surgery (severity: mild-to-moderate; *n* = 28) resolved without residual deficit (Table 4).

##### AAV2-GAD (Unilateral Subthalamic Infusion)

*Motor outcomes.* The phase I study (14) showed a 24% improvement in Off-state UPDRS-III, and a 27% improvement in On-state UPDRS-III after 12 months (pooled data, without statistical comparisons amongst the three dosages) (Table 4). There was a non-significant trend toward UPDRS-IV improvement after the 12-month follow-up (Table 4).

*Other efficacy outcomes.* There were significant reductions in thalamic FDG uptake on PET scan in the treated hemispheres (Table 4). No significant differences were observed in the S&E scale, LEDD, or neuropsychological tests (Table 4).

*Safety data.* There were three serious AEs: a severe freezing episode, an exacerbation of chronic obstructive pulmonary disease, and an arthroscopic knee procedure. All of the reported AEs were transient and classified as unrelated to treatment (Table 4).

#### LV-TH/CH1/AADC (ProSavin)

A cohort of 15 male patients received LV-TH/CH1/AADC through stereotactic intraputaminal bilateral infusions, within the context of one phase I/II open-label, dose-escalation study (18), and subsequent long-term follow-up study of the same cohort (19) (Tables 1, 3, 4).

##### Motor Outcomes

There was an improvement in the Off-state UPDRS-III at 12 months (11.8 points) (18) and at long-term follow up (8.2 points) without a significant difference between the dose cohorts (19). No significant improvements in the On-state UPDRS-III were observed (Table 4). There was a two point improvement in the UPDRS-IV score at 12 months (18), which was not confirmed in the long-term follow-up study (19). No significant differences in mean dyskinesia rating scale scores were reported.

##### Other Efficacy Outcomes

There was an improvement in UPDRS-II (Off-state: 4 points; On-state: 2 points) and PDQ-39 (5.7 points) at 6 months (18). However, these findings were not confirmed during follow-up (19). No significant changes were observed in LEDD, neuropsychological tests, or 18F-DOPA PET uptake in either study.

##### Safety Data

There were three serious AEs: dyskinesia, acute psychosis, and an unspecified nervous system disorder. The safety profile was similar across all dose cohorts. Two deaths were reported after 4 and 6 years and were considered unrelated to the treatment (Table 4).

#### AAV2-GDNF

A total of 13 participants (10 males and 3 females) received AAV2-GDNF through MRI-guided intraputaminal bilateral infusions within the context of a phase I open-label, dose-escalation study (20) (Tables 1, 3, 4).

##### Motor Outcomes

No significant changes were reported in the On-state or Off-state UPDRS-III after 18 months (Table 4). No significant changes were reported in UPDRS-IV (Table 4).

##### Other Efficacy Outcomes

No significant changes were reported in UPDRS-I and -II scores, or LEDD, except for an improvement in Off-state UPDRS-II after 1 month of follow-up. *Post-hoc* analysis showed significant increase in 18F-DOPA putaminal uptake after 6 and 18 months of follow-up (median increase 36% and 54%, respectively; Table 4).

##### Safety Data

One serious AEs related to the surgical procedure was reported: scalp wound dehiscence that required debridement. No other AEs related to AAV2-GDNF or to the surgical procedure were reported (Table 4).

#### AAV2-NRTN

A total of 79 participants (56 males and 23 females) received AAV2-NRTN through stereotactic intraputaminal bilateral infusions (*n* = 50) or intraputaminal plus substantia nigra (SN) bilateral infusions (*n* = 29), within the context of 2 phase-I open-label studies (21, 23), 2 phase II double-blind RCTs (22, 24), and 1 observational long term follow-up study (25) (Tables 1, 3, 4). Three additional studies reported post-mortem data from participants treated with bilateral putamen (*n* = 5) or bilateral putamen plus SN (*n* = 1) AAV2-NRTN infusions (26–28).

##### AAV2-NRTN (Bilateral Putamen Infusion)

*Motor outcomes.* There was a 36% UPDRS-III (Off-state) improvement at 12 months in the phase I open-label trial (21), a greater improvement in active vs. sham group (33% vs. 11%) in the phase II RCT at 18 months (22). The 5-year long-term follow-up, however, demonstrated mixed results, ranging from a 3.2% worsening to a 14.9% improvement (25) of UPDRS III Off-state scores (Table 4). No significant differences in On-state UPDRS-III were reported (Table 4). Three studies (21, 22, 25) reported a significant increase in On-time without troublesome dyskinesia ranging from 1.5 to 2.7 h/day with a trend toward greater improvement with higher doses (Table 4), and a significantly higher improvement in the active vs. sham group (22). One study (21) showed a significant decrease in UPDRS-IV score (18%) (Table 4). Changes in Off-time were not significant in any of the studies (Table 4).

*Other efficacy outcomes.* The RCT (22) showed a greater improvement in the UPDRS-II (2.6 points) and PDQ-39 (6.9 points) in the active vs. sham groups at 12 months. No significant changes were reported on any of the cognitive tests or on 18F-DOPA PET (Table 4).

*Safety data.* Nine AEs were considered possibly related to AAV2-NRTN: psychosis, dizziness, insomnia, altered mood, and dyskinesia. Other transient AEs related to surgery were equally represented in both active and sham groups (Table 4). Two deaths and three cases of cancer were observed but considered unrelated to the treatment.

##### AAV2-NRTN (Bilateral Putamen and Substantia Nigra Infusion)

*Motor outcomes.* The phase I open-label, dose-escalation trial reported an improvement in the Off-state UPDRS-III at 12 months (5 points) and at 24 months (5.5 points) (23), while the phase-IIb RCT found no significant difference in UPDRS-III between active vs. sham group at 15–24 months (24) (Table 4). No data on On-state UPDRS-III were reported and no formal motor assessment data was provided in the 3-year follow-up study (25). There was a 1.1–2.3 h per day decrease in Off-time (23, 24). The phase-IIb RCT showed that this improvement, as well as the amelioration in the UPDRS-IV score, was significantly greater in the active vs. sham group (24) (Table 4). No significant changes in On-time were reported and no data were reported on motor fluctuations in the 3-year follow up (25) (Table 4).

*Other efficacy outcomes.* No significant differences were reported in the PDQ-39 (23, 24), UPDRS-II, or non-motor symptom questionnaire (24) (Table 4).

*Safety data.* There were seven serious AEs: two intracranial hemorrhages (mild symptoms), two coronary artery disease, an acute stroke, bradycardia, and orthostatic hypotension. All of the reported AEs were transient, classified as unrelated to the study drug, and resolved with no residual deficit (Table 4).

##### AAV2-NRTN (Post-Mortem Studies)

Three studies (26–28) reported post-mortem data from six participants treated with AAV2-NRTN (*n* = 5 putaminal infusion, *n* = 1 putaminal and SN infusion), evaluating target structure coverage, NRTN expression, and TH expression (Tables 1, 3, 4). Target coverage ranged from 8.5 to 15% of the putaminal volume, with NRTN expression limited to the transfected regions. TH expression, a surrogate measure for dopamine synthesis capacity, was limited to 2.2–13.4% of the total putaminal volume. The SN coverage in the case treated with putaminal and SN infusion was 56.9–66.6%, with a TH expression of 1.7–3.3 times higher than PD controls. The persistence of recombinant viral genome 8 and 10 years post-surgery, (via *in situ* hybridization) was lower than expected from murine models (28).

### Huntington Disease

A total of 40 participants diagnosed with HD received active treatment and 12 received placebo as part of a study investigating CNTF (*n* = 1 study) (29) and another on ASO-HTT_Rx_ (*n* = 1 study) (30). In the CNTF study, capsules containing polymer-encapsulated BHK cell lines were implanted in the right lateral ventricle and in the ASO-HTT_Rx_ trial intrathecal infusions were used to deliver the study drug (Tables 2, 5, 6).

**Table 5:** Reviewed articles—sample characteristics and endpoints—other diseases.

**Study**	**Dose and volume delivered**	**Follow-up (months)**	**Primary endpoint**	**Other endpoints**
**HUNTINGTON DISEASE—CNTF**/**RIGHT VENTRICLE IMPLANTATION**				
Bloch et al. (29)	0.15–0.5 μg CNTF/day	24	Safety and tolerability	Feasibility assessment, TFC, UHDRS
Open-label/Phase I	Volume not recorded			Changes in striatal 18F-Ldopa uptake
**HUNTINGTON DISEASE—ASO-HTTRx**/**INTRATHECAL INFUSION**				
Tabrizi et al. (30)	10 mg (3 pts)	7–15	Safety and tolerability	HTTRx pharmacokinetics (CSF)
Double-blind RCT/Phase I/IIa	30 mg (6 pts) 60 mg (6 pts) 90 mg (9 pts) 120 mg (10 pts) Volume not recorded			Cognitive and psychiatric function Correlation between UHDRS and mutant HTT concentration
**AADC DEFICIENCY—AAV2-AADC**/**BILATERAL PUTAMEN**				
Hwu et al. (31)	1.6 × 10^11^ vg−320 μL	9–24	Safety and tolerability	Motor function, mood, and oculogyric crises
Open-label/Phase I				Changes in putaminal 18F-Ldopa uptake
Chien et al. (32) Open-label/Phase I/II	1.8 × 10^11^ vg−320 μL	24	Effect on motor development and changes in CSF HVA and 5-HIAA	Surgical complications and adverse events for 12 months
Kojima et al. (33)	2 × 10^11^ vg−200 μL	24	Safety and tolerability	Motor and cognitive functions
Open-label/Phase I/II				Changes in putaminal FMT-PET uptake
**AADC DEFICIENCY—AAV2-AADC**/**BILATERAL SN AND VTA**				
Pearson et al. (34) Open-label/Phase I	8.3 × 10^11^ vg (3 pts)−160 μL 2.6 × 10^12^ vg (4 pts)−160 μL	7–38	Safety and biological AADC activity (CSF metabolites concentrations, changes in 18F-Ldopa uptake)	Motor improvement (GMFM-88), frequency of oculogyric crisis

*AADC, aminoacid decarboxylase; AAV2, Adeno-Associated Viral Vector 2; CNTF, Ciliary Neurotrophic Factor; CSF, Cerebrospinal Fluid; FDOPA PET, fluorodopa PET scan; FMT-PET, 18F-fluoro-L-m-tyrosine PET scan; GMFM-88, Gross Motor Function Measure-88; HTTRx, Antisense Oligonucleotide Targeting HTT mRNA; 5-HIAA, 5-hydroxyindoleacetic acid; HVA, Homovanillic acid; NA, Not Applicable; NR, Not Reported; pts, patients; SN, Substantia Nigra; TFC, Total Functional Capacity Scale; UHDRS, Unified Huntington's Disease Rating Scale; vg, vector genomes; VTA, Ventral Tegmental Area*.

**Table 6:** Reviewed articles—efficacy and safety—other diseases.

**Study**	**Efficacy**	**Adverse events**
**HUNTINGTON DISEASE—CNTF**/**RIGHT VENTRICLE IMPLANTATION**		
Bloch et al. (29)	- Electrophysiological changes in three patients	- Three major depressions after trial end, not directly related to CNTF
Open-label/Phase I	- No significant improvement in TFC or UHDRS	
	- No significant changes in metabolic striatal activity	
**HUNTINGTON DISEASE—ASO-HTTRx**/**INTRATHECAL INFUSION**		
Tabrizi et al. (30) Double-blind RCT/Phase I/IIa	−20–63% (dose-dependent) reduction of CSF mHTT concentration in active cohorts vs. 10% increase in placebo - No significant improvement in functional, cognitive, psychiatric, and neurologic clinical outcomes	- Mild/moderate, transient, AEs (most related to lumbar puncture) - No significant difference between active and sham groups
	- Greater ventricular volume increase in 90 and 120 mg cohorts vs. placebo	
	- Correlation between UHDRS improvement and decreased CSF mHTT concentration	
**AADC DEFICIENCY—AAV2-AADC**/**BILATERAL PUTAMEN**		
Hwu et al. (31)	- AIMS and PDMS-2 motor score improvement in all patients	- Transient choreic dyskinesia in all patients
Open-label/Phase I	- CDIIT improvement in all patients	- Transient increase in apneic episodes in one patient
	- Increased putaminal 18F-Ldopa uptake uptake in three patients	
Chien et al. (32)	- PDMS-2 scores were increased (median: 62 points)	- Transient dyskinesia in all patients (resolved with risperidone)
Open-label/Phase I/II	- HVA CSF concentration increased (median: 25 nmol/L)	−31 treatment-related AEs, one severe (life-threatening hyperpyrexia)
	- No significant change in 5-HIAA CSF concentration	- One death due to Influenza B encephalitis, unrelated to treatment
Kojima et al. (33)	- AIMS motor scores improvement in all patients	- Transient choreic dyskinesia in all patients
Open-label/Phase I/II	- Cognition improvement	- One asymptomatic subdural hemorrhage related to surgery
	- Appearance of FMT-PET uptake in all patients	
**AADC DEFICIENCY—AAV2-AADC**/**BLATERAL SN AND VTA**		
Pearson et al. (34)	- Appearance of FMT-PET uptake in all patients	- Transient worsening of irritability and sleep disturbance
Open-label/Phase I	- HVA CSF concentration increased in all patients	- Transient choreic dyskinesia in all patients
	- OGCs and GMFM-88 improved in all patients	- One sudden death at home (cause of death unknown, judged to be attributable to the primary disease)

*AADC, aminoacid decarboxylase; AAV2, Adeno-Associated Viral Vector 2; AIMS, Alberta Infant Motor Score; CDIIT, Comprehensive Developmental Inventory for Infants and Toddlers; CNTF, Ciliary Neurotrophic Factor; CSF, Cerebrospinal Fluid; FMT-PET, 18F-fluoro-L-m-tyrosine PET scan; GMFM-88, Gross Motor Function Measure; HTTRx, Antisense Oligonucleotide Targeting HTT mRNA; HVA, Homovanillic acid; mHTT, mutant HTT; NR, Not Reported; OGCs, Oculogyric Crises; PDMS-2, Peabody Developmental Motor Scores-2; SN, Substantia Nigra; TFC, Total Functional Capacity Scale; UHDRS, Unified Huntington's Disease Rating Scale; vg, vector genome; VTA, Ventral Tegmental Area*.

#### CNTF

A total of six participants (three males and three females) were involved in a phase I open label study (29) and underwent implantation of capsules containing cells engineered to synthesize CNTF. Capsules were exchanged every 6 months for a total of four capsules (Tables 2, 5, 6).

##### Feasibility

The study demonstrated the feasibility of the procedure. Implantations were well tolerated, and capsule devices were able to remain in the CSF for months and be removed without significant difficulty. However, the capsules did not demonstrate reliable drug delivery: though all capsules released a detectable level of CNTF in the perioperative period, only 11 of 24 explanted capsules still secreted a detectable level after 6 months.

##### Motor Outcomes

No significant changes were reported in TFC or UHDRS. However, a trend toward a UHDRS amelioration was observed in those participants with capsules that were still actively secreting (Table 6).

##### Other Efficacy Outcomes

The study did not report any significant increase in striatal uptake by FDG-PET (Table 6). Three participants showed an improvement of electrophysiological parameters (recovery of normal range values for either somatosensory evoked potentials or for intracortical inhibitory response to motor cortex transcranial magnetic stimulation) (Table 6).

##### Safety Data

No AEs were considered related to CNTF or the surgical procedure. Following trial conclusion, three subjects displayed mood deterioration, in two cases requiring hospitalization; these AEs were most likely linked to the lack of any future therapeutic options and not directly related to CNTF (Table 6).

#### ASO-HTT_Rx_

A total of 46 participants (*n* = 12 placebo, *n* =34 active with five therapeutic dosages) received 4 monthly intrathecal infusions of the ASO HTT_Rx_via lumbar spinal tap in a phase I/IIa, double-blind, dose-escalation RCT. Intrathecal infusions were performed every 4 weeks for four total doses and participants were followed-up for 7–15 months (30) (Tables 2, 5, 6).

##### ASO-HTT_Rx_ Pharmacokinetics

ASO-HTT_Rx_ was measurable in the CSF at therapeutic doses ≥30 mg, and CSF concentrations increased with escalating doses. Peak plasma concentrations were reached within 4 h and declined to <30% of the peak by 24 h. No accumulation of ASO-HTT_Rx_ was detected within the CSF or plasma was observed (Table 6).

##### Clinical Effficacy

No significant changes in UHDRS or HD Cognitive Assessment Battery were reported. However, a *post-hoc* analysis observed a correlation (no statistical data available) between CSF mutant HTT (mHTT) reduction and improvement in composite UHDRS (Table 6).

##### Other Outcome Measures

The study reported a dose-dependent decrease in mHTT concentration in the CSF ranging from −20/−42% in the active group as compared to a 10% increase in the placebo group (Table 6). Dose and time-dependent increases in ventricular volume on MRI in the highest dose groups were described (Table 6) as well as transient elevations of CSF neurofilament light chain (Table 6).

##### Safety Data

There was one serious AE: post-lumbar puncture headache. Mild-to-moderate AEs were reported in 98% of participants, most related to the lumbar puncture and equally represented in both the active and the placebo group (Table 6). Only 6% of AEs were considered treatment-related; all were transient and resolved with no residual deficit (Table 6).

### AADC Deficiency

Twenty-seven participants (14 males and 13 females) received AAV2-AADC through intraputaminal bilateral infusions (*n* = 20) or SN and ventral tegmental area (VTA) (*n* = 7), in 2 phase I/II open-label studies, one phase I open-label study, and one compassionate use study, and were followed-up for 9–38 months (31–34) (Tables 2, 5, 6).

#### Motor Outcomes

All participants reported an amelioration in the Alberta Infant Motor Score, in the Peabody Developmental Motor Scores-2, or in the GMFM-88: Gross Motor Function Measure-88. There was a trend toward higher improvement in younger participants (no statistical data available) (Table 6). Swallowing and respiration were improved, dystonia attacks resolved, and oculogyric crises decreased in frequency and/or severity to varying degrees between individuals.

#### Mental Status

Cognitive scores (Kyoto Scale of Psychological Development-Cognitive-Adaptation and Language-Sociality, or Comprehensive Developmental Inventory for Infants and Toddlers) improved in all participants. However, across these studies, the greatest improvements were seen in the participants with a mild to moderate disease presentation (Table 6).

#### Other Efficacy Data

When available, imaging data showed more prominent increase in radiotracer uptake in regions that correlate with areas of infusions. FMT-PET putaminal uptake (33) or 18F-DOPA PET midbrain, putaminal, and caudate uptake (34) passed from absent to bilaterally present with high signal intensity, after up to 2-year follow-up, while in the study from Hwu and coll (31) three out of four participants showed an increased 18F-DOPA uptake, as compared to pre-treatment imaging (Table 6). CSF catechol analysis demonstrated levels that trended toward increased dopamine metabolites across these studies, but degree of change in serotonin metabolites and levodopa varied significantly across individuals both before and after treatment. Qualitative improvements in feeding, mood, autonomic functions, and sleep were also reported by majority of caregivers for these pediatric participants (31–34).

#### Safety Data

Three serious AEs were reported: one asymptomatic subdural hemorrhage related to the surgical procedure (33), one life-threatening hyperpyrexia (32), and one transient increase of apneic episodes (31). Two deaths due to Influenza B encephalitis (32) or to unknown cause (34) were considered unrelated to treatment. All participants experienced transient choreic movements weeks to months after surgery to varying degrees and most were managed with medication adjustments similar to what has been seen in the PD AAV2-AADC studies (Table 6).

### Ongoing Studies

Our search on ClinicalTrials.gov retrieved nine ongoing studies on PD, five on HD, two on multiple system atrophy (MSA), two on AADC deficiency, and one on progressive supranuclear palsy (PSP) (Table 7).

**Table 7:** Ongoing trials.

**NCT**	**Target population**	**Compound delivered**	**Route of administration**	**Sample size**	**Completion date**	**Active/Placebo**	**Primary outcome**	**Secondary outcomes**
**PARKINSON DISEASE**								
NCT01621581 Phase I	PD duration >5 years Age >18	AAV2-GDNF	Intrastriatal infusion (bilateral putamen)	25	2/2022	Non-randomized	Safety and tolerability	Clinical response (laboratory, imaging, and clinical scales)
	UPDRS-III ≥30							
NCT01856439 Phase I/II	PD pts enrolled in prior study	ProSavin (LV-TH, AADC-CH1)	Intrastriatal infusion (bilateral putamen)	15	05/2022	N/A	Long term safety and tolerability	Clinical response (UPDRS-III, motor fluctuations, PDQ-39)
NCT01973543 Phase I	PD duration >5 years UPDRS-III 25–60 Age 40–70	VY-AADC01 (AAV2-AADC)	Intrastriatal infusion (bilateral putamen)	15	01/2020	Non-randomized	Safety and tolerability	PD symptoms (motor and non-motor), PET changes in AADC distribution and expression
NCT03065192 Phase I	PD duration >5 years UPDRS-III ≥25 Age 40–75	VY-AADC01 (AAV2-AADC)	Intrastriatal infusion (bilateral putamen)	16	12/2021	N/A	Safety and tolerability	Change in PD medications, changes in motor scores, dyskinesias, mood, cognitive performances
NCT03562494Phase II—Not Recruiting	PD duration >4 years Age 40–75	VY-AADC02 (AAV2-AADC)	Intrastriatal infusion (bilateral putamen)	85	12/2022	Randomized, Sham surgery-controlled	Change in On-time (diary)	Change in UPDRS-II and -III score, AEs
	Off-time ≥3 h							
NCT03720418 Phase I/II	UPDRS-III 30–60 Age 30–70	OXB-102 (LV-TH, AADC-CH1)	Intrastriatal infusion (bilateral putamen)	30	12/2031	Randomized, Sham surgery-controlled	Safety and tolerability	Change in UPDRS scores in On and Off, changes in motor fluctuations and dyskinesias
NCT03976349 Phase I	PD duration >7 years Hoehn & Yahr stage ≤3 Age 35–80	BIIB094 (ASO-LRRK2)	Intrathecal infusion	82	12/2022	Randomized, Placebo-controlled, dose-escalation	Safety and tolerability	Pharmacokinetic profile of BIIB094
NCT04127578 Phase I/II	PD with at least one pathogenic GBA1 mutation Hoehn and Yahr stage ≥3 Age 35–80	PR001A (AAV9-GBA1)	Intrathecal infusion	12	06/2027	Non-randomized	Safety and tolerability (immunogenicity)	Changes in glycolipid, GCase levels and activity in blood and CSF
NCT04167540 Phase Ib	Mild/moderate PD with duration <5 years (*n* = 6)	AAV2-GDNF	Intrastriatal infusion (bilateral putamen)	12	06/2026	Non-randomized	Safety and tolerability	MDS-UPDRS-III, non-motor symptom scale, brain dopaminergic cells integrity (DaTscan)
	Moderate/severe PD duration >3 years (*n* = 6)							
	Hoehn and Yahr stage ≤3							
	Age 35–75							
**MULTIPLE SYSTEM ATROPHY**								
NCT04680065 Phase I	Probable or possible MSA-P Age 35–75	AAV2-GDNF	Intrastriatal infusion (bilateral putamen)	9	01/2024	Randomized, double-blinded, placebo-controlled	Safety and tolerability	UMSARS I and II; QoL measures;
NCT04165486 Phase I	Probable or possible MSA-P or MSA-C Age 40–70	BIIB101 (ASO-SNCA)	Intrathecal infusion	34	07/2022	Randomized, dose-escalation, placebo-controlled	Safety and tolerability	Pharmacokinetics profile
**HUNTINGTON DISEASE**								
NCT03761849 Phase III	CAP score >400 Independence Scale ≥70Age 25–65	RG6042-tominersen (ASO-HTT)	Intrathecal infusion	909	07/2022	Randomized, Placebo-controlled	UHDRS composite and TFC	Other UHDRS components, clinical global impression, AEs, MoCA, C-SSRS, Pharmacokinetics markers, CSF mHTT and neurofilament light chain, MRI brain volumes
NCT04000594 Phase I	CAP score >400 Independence Scale ≥70 Age 25–65	RG6042-tominersen (ASO-HTT)	Intrathecal infusion	20	12/2021	Non-randomized	Pharmacokinetics of RG6042 in CSF and plasma; CSF mHTT	AEs, C-SSRS, incidence and titers of antidrug Abs, concentration RG6042 in urine
NCT03225833 Phase Ib/IIa	HD stage I or II SNP rs362307 Age 25–65	WVE-120101 (ASO-HTT)	Intrathecal infusion	60	12/2020	Randomized, Placebo-controlled	Safety and tolerability at 210 days	Pharmacokinetics measures in plasma, Pharmacodynamics measures in CSF (mHTT protein), UHDRS TFC
NCT03225846 Phase Ib/IIa	HD stage I or II SNP rs362331 Age 25–65	WVE-120102 (ASO-HTT)	Intrathecal infusion	60	12/2020	Randomized, Placebo-controlled	Safety and tolerability at 210 days	Pharmacokinetics measures in plasma, Pharmacodynamics measures in CSF (mHTT protein), UHDRS-TFC
NCT04120493 Phase I/II	TFC 9–13 40CAG repeatsPutamen V ≥2.5 cm^3^ Caudate V ≥2 cm^3^ Age 25–65	AMT-130 (rAAV5-miHTT)	Intrastriatal infusion (bilateral putamen and caudate)	26	05/2026	Randomized, Sham surgery-controlled	Safety and tolerability	CSF biomarkers (vector DNA and miRNA exp), biofluid and imaging biomarkers, UHDRS, HD-CAB, HDQLIFE, HADS, Quantitative motor testing
**AADC D****EFICIENCY**								
NCT02926066 Phase II	Definite diagnosis of AADC deficiency Age 2–6	AAV2-AADC	Intrastriatal infusion (bilateral putamen)	12	01/2022	Non-randomized	Efficacy (changes in CSF neurotransmitter concentrations and PDMS-II) at 13 months	Safety and AEs, pharmacokinetics, changes in FDOPA-PET scan.
NCT01395641 Phase I/II	Definite diagnosis of AADC deficiency Age >2 years	AAV2-AADC	Intrastriatal infusion (bilateral putamen)	10	12/2020	Non-randomized	Efficacy (changes in CSF neurotransmitter concentrations and PDMS-II) at 12 months	Safety and AEs, pharmacokinetics, changes in FDOPA-PET scan
**PROGRESSIVE SUPRANUCLEAR PALSY**								
NCT04539041 Phase I	PSP diagnosis for <5 years PSPRS score <40, MoCA > 17	NIO752 (ASO-MAPT)	Intrathecal infusion	64	10/2023	Randomized, Placebo-controlled	Safety and tolerability at 1 year	Pharmacokinetic measures in CSF and plasma
	Age 40–75							

*AADC, aminoacid decarboxylase; AAV2, Adeno-Associated Viral Vector 2; AAV5, Adeno-Associated Viral Vector 5; AAV9, Adeno-Associated Viral Vector 9; AEs, Adverse Events; ASO, Antisense Oligonucleotide; CAP, CAG Age Product; GBA1, glucocerebrosidase gene; GCase, glucocerebrosidase; GDNF, Glial cell line-Derived Neurotrophic Factor; HADS, Hospital Anxiety and Depression Scale; HD, Huntington Disease; HD-CAB, Huntington's Disease Cognitive Assessment Battery; HDQLIFE, Huntington's Disease Quality of Life scale; LRRK2, leucine-rich repeat kinase 2; LV, Lentivirus; MAPT, Microtubule-Associated Protein Tau; MDS-UPDRS, Movement Disorder Society-Unified Parkinson's Disease Rating Scale; mHTT, mutant HTT protein; miHTT, microRNA for HTT; MoCA, Montreal Cognitive Assessment; MRI, Magnetic Resonance Imaging; PD, Parkinson Disease; PDMS-II, Peabody Developmental Motor Score-2; PDQ-39, Parkinson's disease Questionnaire 39; PSP, Progressive Supranuclear Palsy; PSPRS, Progressive Supranuclear Palsy Rating Scale; SNc, Substantia Nigra pars compacta; SNP, Single Nucleotide Polymorphism; TH, Tyrosine Hydroxylase; UHDRS-TFC, Unified Huntington's Disease Rating Scale-Total Functional Capacity; UMSAR, Unified multiple System Atrophy Rating Scale; UPDRS, Unified Parkinson's Disease Rating Scale*.

In PD, three studies will evaluate AAV2-AADC (one of which active but not recruiting due to asymptomatic MRI abnormalities seen at 12 months post-surgery), two AAV2-GDNF (one of them very close to completion), and one LV-TH/CH1/AADC (ProSavin) (Table 7). The remaining three studies will evaluate new compounds: OXB-102, an optimized version of ProSavin, expressing the same enzymes but with increased DA production (35); BIIB094, an ASO developed to reduce LRRK2 protein levels with repeated intrathecal infusions; and AAV9-GBA (PR001A), a vector-mediated delivery of a functional copy of GBA1 with the aim of constitutively increasing the GCase activity following a single infusion of the study drug. A total of 292 PD participants will take part in these studies.

In MSA, a randomized, double-blind, placebo-controlled phase I study is planned to enroll a total of nine participants (six receiving treatment and three receiving placebo) (Table 7). This first-in-human study will utilize MRI-guided bilateral intraputaminal infusions of AAV2-GDNF with the aim of restoring the putaminal GDNF deficiency found in post-mortem tissue of MSA patients. Safety and tolerability will be the primary endpoint for this phase I study. A phase I randomized, placebo-controlled, dose-escalation study will enroll 34 participants to receive repeated intrathecal infusions of ASO-SNCA (BIIB101) or placebo. The primary objective is safety and tolerability, and pharmacokinetic profile of BIIB101 is a secondary objective.

In PSP, a phase-I, multi-center, double-blind, placebo-controlled, multiple dose-escalation study will enroll 64 participants randomized to receive NIO752, an ASO developed to reduce the expression of Tau protein (Table 7). Patients will be divided into six cohorts and randomized to receive intrathecal NIO752 or placebo in a 3:1 ratio four times over 3 months. The primary outcome includes safety measures. Secondary outcomes will focus on pharmacokinetics and concentrations of NIO752 in the blood plasma and CSF.

In HD, four studies will evaluate the efficacy of intrathecally-infused ASOs targeting HTT mRNA (RG6042, WVE-120101, and WVE-120102), with the aim of reducing clinical meaures of HD progression and accumulation of the mutant HTT in CSF (Table 7). One study will evaluate rAAV5-miHTT (AMT-130) in a randomized, double-blind, dose-escalation, sham-surgery controlled study in early manifest HD participants. The primary outcome will be safety, but CSF levels of AMT-130, mutant huntingtin protein, and other biomarkers will be measured in addition to other disease-specific clinical scales. Safety and tolerability are the primary outcome. A total of 1,075 HD participants will take part in these studies.

In AADC deficiency, two studies will evaluate AAV2-AADC. The primary outcome measure will be biological and clinical efficacy (CSF neurotransmitter metabolite profile and motor scales). Secondary outcomes will include safety, pharmacokinetics profile, and changes in 18F-DOPA PET signal following gene transfer. A total of 22 participants are anticipated to be enrolled in these studies.

## Discussion

Multiple clinical trials have examined the safety and preliminary efficacy of gene therapy for PD, HD, and AADC deficiency using complementary DNA sequences encapsulated into viral vectors (AAV2, LV), polymer-encapsulated cell lines engineered to synthesize neurotrophic factors (CNTF), or ASOs. Ongoing clinical trials are also exploring alternative viral vectors (AAV5, AAV9), recombinant genes (OXB-102, GBA1, and HTT), and novel antisense oligonucleotides (LRRK2, HTT, SNCA, and MAPT) for the treatment of several neurodegenerative disorders with a high unmet need to develop disease-modifying therapies.

Several PD gene therapy studies have demonstrated relative safety in multiple clinical trials aimed at restoring the synthesis of dopamine (8–13, 18, 19), enhancing the production of trophic factors (20–25), or modifying the functional interaction between different nodes of the basal ganglia (14–17). There was a variable improvement in the Off-state UPDRS-III with gene therapy approaches aiming at providing dopamine restoration (AAV2-AADC and LV-TH-CH1-AADC) or neuromodulating the basal ganglia network (AAV2-GAD). A variable extent of Off-state UPDRS-III reduction or stability of scores were also observed with gene therapy strategies based on enhancing the production of neurotrophic factors (AAV2-NRTN and AAV2-GDNF). Still, post-mortem studies (26–28) demonstrated limited target coverage in the putamina. These findings indicate the potential for greater clinical benefit by increasing infusion volumes to expand the amount of putaminal tissue exposed to the study drug with current volumes of administration now 4–5 times higher than earlier studies (36). The use of neuroimaging biomarkers, mostly FMT and 18F-DOPA PET, confirmed an increase in the AADC activity or dopamine storage terminal capacity in the AAV2-AADC and AAV2-GDNF trials (8–12, 20). Functional reorganization of the pathways linking the STN with cortical motor regions with AAV2-GAD was demonstrated through metabolic changes by FDG PET following treatment (14, 16, 17). The relative improvement demonstrated by neuroimaging suggests its employment as a key endpoint for gene therapy studies; however, the correlation between imaging and clinical change has been inconsistent and remains a challenge for the field. Ongoing trials aim to evaluate improved formulations of symptomatic and neurotrophic approaches and to test innovative recombinant genes or ASOs to treat specific genetic subtypes of PD, such as GBA1- and LRRK2-associated PD.

In HD, the ASO-HTT_Rx_ trial showed that monthly intrathecal delivery of the ASO was safe and tolerable, and demonstrated a dose-dependent reduction of CSF mHTT that persisted 2 months post dose (30). The study was not designed to assess clinical efficacy, but a *post-hoc* analysis suggested a correlation between improvement in composite UHDRS and CSF mHTT reduction. The ongoing multinational phase III study of the same RG6042 ASO (known as tominersen) will assess 909 participants, comparing clinical progression in those treated with RG6042 vs. placebo controls. Tominersen is a non-allele-specific ASO that reduces expression of both mutant and wild type HTT mRNA. Two phase Ib/IIa RCTs, designed to assess safety, tolerability, and pharmacokinetics, employ allele-specific ASOs, WVE-120101, and WVE-120102. These compounds target single nucleotide polymorphisms (SNPs) found in selected variants of the mHTT gene, thereby knocking down mHTT while preserving wtHTT expression. Though preclinical studies in non-human primates showed ASO target engagement in deep subcortical brain regions following lumbar intrathecal infusion (37), this has not yet been demonstrated in humans. Two studies, one ongoing and another planned (38) will assess the safety of direct subcortical administration of AAV-mediated siRNAs designed to persistently reduce mHTT expression after a single intracranial procedure.

Gene therapy studies have demonstrated to be relative safety in four AADC deficiency trials aimed at restoring catecholaminergic synthesis by constitutively enhancing AADC enzymatic capacity following a single infusion of AAV2-AADC. In the four reported studies, over a dozen serious AEs were reported, however, the majority of these events were expected following enhanced AADC activity (dyskinesia, orofacial dyskinesias) and other events have been reported with underlying disease progression (apneic episodes, feeding difficulties). Intriguingly, all studies reported promising results with amelioration of motor and cognitive performances, in particular in younger participants with milder baseline impairment (31–34). Notably, dysphagia and respiratory function improved, as well as dystonia and oculogyric crises; all of which are disease manifestations that have a high impact on quality of life as reported by caregivers of children with AADC deficiency. Further refinement of gene therapy delivery by directly targeting catecholaminergic midbrain nuclei, as compared to the downstream putaminal targets previously investigated, has recently demonstrated to be a promising approach to improve the magnitude of clinical effect in these neurodevelopmentally compromised children (34).

**Figure 2:**
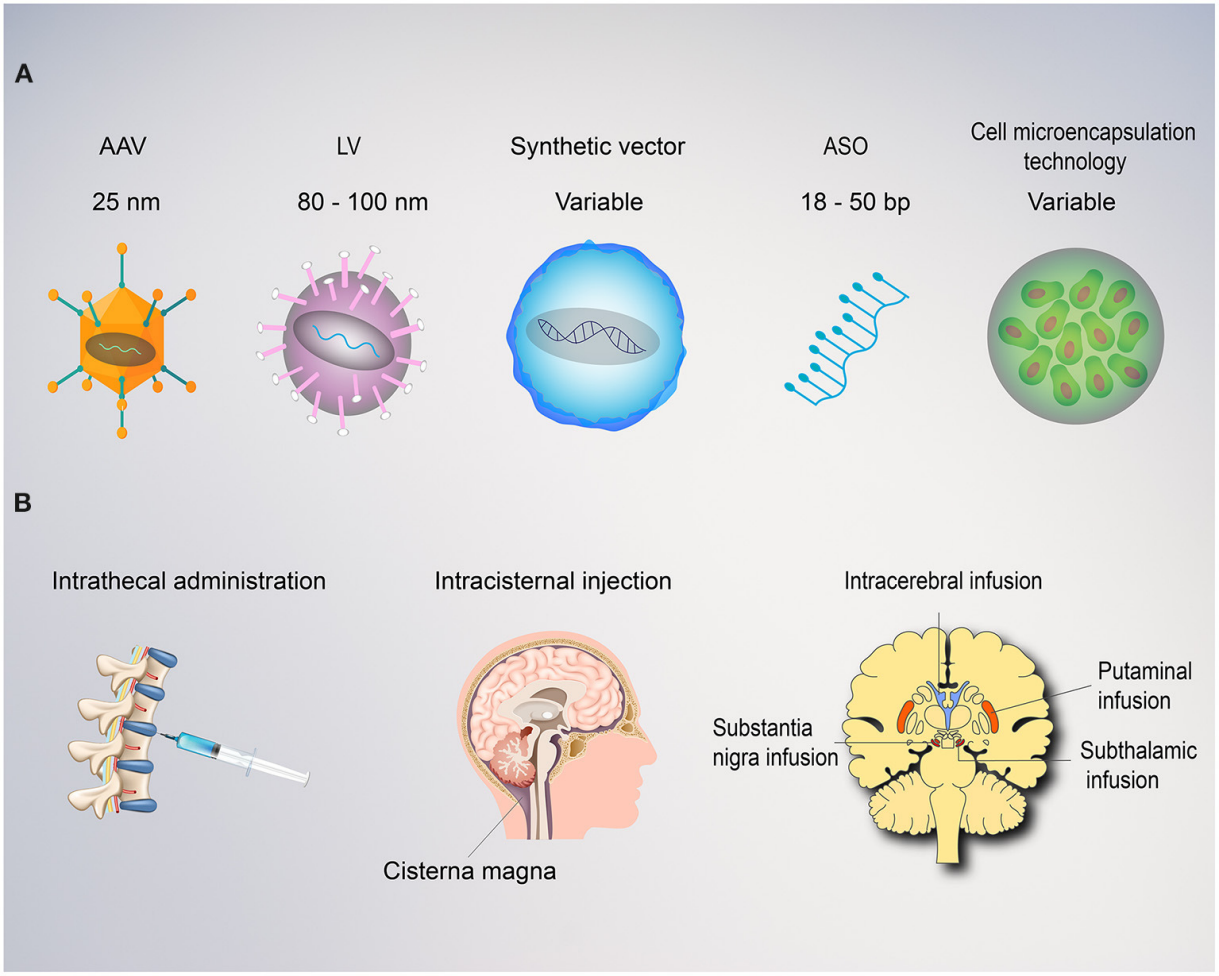
Vectors and route of administration for gene therapy in movement disorders. **(A)** Both viral and non-viral vectors can be employed for gene therapy. Adeno associated viruses (AAVs) are relatively small with ~4.0 kb packaging capacity and lower immunogenic potential when delivered directly to the brain. AAVs replicate primarily as episomes within the nucleus and rarely integrate into the genome with a low risk of insertional mutagenesis. Lentiviruses (LV) are larger with an 8.5 kb carrying capacity. LVs utilize reverse transcriptase, allowing the transgene to integrate into the host genome, providing stable, long-term transgene expression, but also carries a higher risk of insertional mutagenesis. Synthetic vectors are characterized by lower immunogenicity and larger carrying capacity, but poor delivery efficiency. Antisense oligonucleotides (ASO) are short, single-stranded synthetically derived oligonucleotides. In application for movement disorders, these are typically designed to bind mRNA to interfere with the mechanism of protein translation and thereby reduce levels of aberrant proteins. Cell microencapsulation technology employ cells genetically engineered to secrete proteins and encapsulated into an immunoisolating material that makes them suitable for transplantation. **(B)** The route of administration is a critical factor for the success of any movement disorders gene therapy approach. Intrathecal, intraventricular, or intracisternal magna injections provide wide distribution of the vector within the central nervous system, but poor diffusion into the deep regions of the target basal ganglia. This approach requires dilution into large volumes of cerebrospinal fluid and delivery of high titers with a consequent increase in cost and risk of immunogenicity. Intraparenchymal delivery ensure precise delivery and optimal vector distribution within the critical structures involved (i.e., putamina, substantia nigra, and subthalamic nucleus).

Multiple factors can influence the efficacy of gene therapy, with the route of administration being one of the most critical. The majority of trials for movement disorders have leveraged intraparenchymal administration to deliver the gene vector directly to the region of interest in the brain (8–25, 29–34). This technique substantially reduces the dose of vector required and reduces potential off-target spread into peripheral tissues, both of which limit the potential immunogenicity (39). In addition to achieving higher vector levels in target target brain regions as compared to intravenous or intrathecal administration routes, studies have also demonstrated intranetwork vector distribution through axonal transport to other brain regions via topographical connections (40, 41). Distribution volume within the target region of the brain can be further enhanced through the use of convection-enhanced delivery (CED), a positive pressure infusion system, to drive the regional spread of vector. This is a critical discovery as volume of coverage and resultant gene expression levels have demonstrated correlation in several humans and non-human studies (9, 13, 42, 43). When a broader delivery is required within the central nervous system, intrathecal, intracerebroventricular, subpial, and intracisternal infusions can also be utilized (Figure 2). Drawbacks to these techniques include higher required doses, lower target tissue levels of vector transduction, as well as more heterogeneous distribution. Finally, intravenous delivery of gene therapy for neurologic disorders are also being explored preclinically. This less invasive mode of delivery is an attractive option with the potential to transfer genes to the entire central nervous system (44). However, its use is limited by the blood brain barrier that requires substantially higher doses, carries the potential for antibody opsonization within the systemic circulation, and substantially increases the risk of immunogenicity and peripheral distribution of the vector (1, 39). Development of less immunogenic capsids and cell-specific enhancers will be critical to safely advance intravenous gene therapies, in particular for neurologic disorders.

In conclusion, multiple phase-I and early phase-II studies have been critical in informing the development of the ongoing generation of gene therapy trials. Simultaneously, advancements in understanding viral and non-viral vector efficiency, specificity, and duration of transgene expression have helped select the most promising approaches to treat movement disorders symptoms or administer putative disease-modifying interventions. Still, several limitations should be considered in the interpretation of data. First, most of the studies are characterized by small sample size and many did not include a control group. Therefore, limited conclusions can be drawn on the impact of the placebo effect in these open-label studies, which is known to be especially strong in surgical/interventional trials. Additionally, although the length of follow-up is adequate to demonstrate the short-term safety profile for all evaluated compounds, there are few studies that examine the longterm safety profile, and no definitive conclusions can be drawn on the longterm clinical efficacy. This is even more significant when considering that neurotrophic factors are expected to manifest their neuroprotective effect over several years. Third, most of the trials involving intraputaminal administration for PD, HD, and AADC deficiency were affected by a substantial variability in target coverage, which have been deemed insufficient in a small number of *post-hoc* analyses and post-mortem immunohistochemical studies. This factor might have been critical in limiting the efficacy of the compounds delivered, and further refinement of delivery techniques are needed to improve reproducibility and consistency of intraparenchymal drug delivery. Fourth, the lack of cerebrospinal fluid biomarkers of PD progression and the preferential inclusion advanced PD patients prevented the ability to test the “disease-modifying” effect of gene therapy in PD. Although the lack of widely validated biomarkers of disease progression continues to represent a limitation in assessing the long-term projected effect of gene therapy in PD, currently ongoing studies are anticipated to provide additional data resulting from the inclusion of a population of early PD patients. Finally, the heterogeneity of study designs limits a direct comparison between the different therapeutic approaches and the possibility of running a meta-analysis.

Improvement in the molecular strategies used to modulate gene expression and target specificity, as well as increasing attention to a precision-medicine approach represent critical opportunities for improvement in upcoming clinical trials and are anticipated to represent the most significant innovations in the future of gene therapy.

## Data Availability Statement

The original contributions presented in the study are included in the article/Supplementary Material, further inquiries can be directed to the corresponding author/s.

## Author Contributions

AM: conception, organization, and execution of research project: writing of the first draft and review, and critique of manuscript. NK: organization and execution of research project: writing of the first draft and review and critique of the manuscript organization and execution of research project, review, and critique of the manuscript. AR: organization and execution of research project, review, and critique of the manuscript. BW and AV: writing of sections of the first draft, review, and critique of the manuscript. CA, GI, and IL: execution of research draft, review, and critique of the manuscript. KB: conception, organization and execution of research project, review, and critique of manuscript. All the co-authors listed above gave their final approval of this manuscript version.

## Conflict of Interest

AM is supported by NIH (KL2 TR001426) and has received speaker honoraria from CSL Behring, Abbvie, and Cynapsus Therapeutics. He has received grant support from Lundbeck. AR has received grant support and speaker honoraria from AbbVie, speaker honoraria from Chiesi Farmaceutici and travel grants from Lusofarmaco, Chiesi Farmaceutici, Medtronic, and UCB Pharma. BW has received grant support from Acadia and Tilray. CA has received travel grants from Zambon and Abbvie, and educational grants from Ralpharma and Neuraxpharm. IL research is supported by the National Institutes of Health grants: 2R01AG038791-06A, U01NS090259, U01NS100610, U01NS80818, R25NS098999, P20GM109025; U19 AG063911-1; 1R21NS114764-01A1; Parkinson Study Group, Michael J. Fox Foundation, Parkinson Foundation, Lewy Body Association, Roche, Abbvie, Biogen, EIP-Pharma, and Biohaven Pharmaceuticals. She was member of the Scientific Advisory Board of a Lundbeck and Corticobasal Degeneration Solutions. She receives her salary from the University of California San Diego and as Chief Editor of Frontiers in Neurology. AV has received grant support from Voyager Therapeutics and is employed by Asklepios BioPharmaceutical, Inc. KB is the founder of Brain Neurotherapy Bio and Voyager Therapeutics, gene companies. He has equity in Brain Neurotherapy Bio, Asklepios BioPharmaceutical, and Voyager Therapeutics. The remaining authors declare that the research was conducted in the absence of any commercial or financial relationships that could be construed as a potential conflict of interest.
