# Supplementary material for: Gene Therapy in Movement Disorders: A Systematic Review of Ongoing and Completed Clinical Trials
Source: Front Neurol. 2021 Apr 6;12:648532. doi: 10.3389/fneur.2021.648532 (PMC8056023; doi:10.3389/fneur.2021.648532)
Supplement: Supplementary file 1 [file Table_1.docx]

**SUPPLEMENTARY TABLE 1. Search terms and strategy**

| **Search number** | | **Search term** | |
| --- | --- | --- | --- |
| #1 | | Parkinson Disease [MeSH Terms] | |
| #2 | | Huntington Disease [MeSH Terms] | |
| #3 | | Ataxia [MeSH Terms] | |
| #4 | | Dystonia [MeSH Terms] | |
| #5 | | Movement Disorders [MeSH Terms] | |
| #6 | | Genetic therapy [MeSH Terms] | |
| #7 | | Case report | |
| #8 | | AAV | |
| #9 | | Adenovirus | |
| #10 | | Adeno | |
| #11 | | Lentivirus | |
| #12 | | ASO | |
| #13 | | Antisense Oligonucleotide | |
| #14 | | Antisense | |
| **Search strategy** | | | |
| #1 AND #6 | #2 AND #9 | #3 AND #11 | #4 AND #13 |
| #1 AND #6 AND #7 | #2 AND #7 AND #9 | #3 AND #7 AND #11 | #4 AND #7 AND #13 |
| #1 AND #8 | #2 AND #10 | #3 AND #12 | #4 AND #14 |
| #1 AND #7 AND #8 | #2 AND #7 AND #10 | #3 AND #7 AND #12 | #4 AND #7 AND #14 |
| #1 AND #9 | #2 AND #11 | #3 AND #13 | #5 AND #6 |
| #1 AND #7 AND #9 | #2 AND #7 AND #11 | #3 AND #7 AND #13 | #5 AND #6 AND #7 |
| #1 AND #10 | #2 AND #12 | #3 AND #14 | #5 AND #8 |
| #1 AND #7 AND #10 | #2 AND #7 AND #12 | #3 AND #7 AND #14 | #5 AND #7 AND #8 |
| #1 AND #11 | #2 AND #13 | #4 AND #6 | #5 AND #9 |
| #1 AND #7 AND #11 | #2 AND #7 AND #13 | #4 AND #6 AND #7 | #5 AND #7 AND #9 |
| #1 AND #12 | #2 AND #14 | #4 AND #8 | #5 AND #10 |
| #1 AND #7 AND #12 | #2 AND #7 AND #14 | #4 AND #7 AND #8 | #5 AND #7 AND #10 |
| #1 AND #13 | #3 AND #6 | #4 AND #9 | #5 AND #11 |
| #1 AND #7 AND #13 | #3 AND #6 AND #7 | #4 AND #7 AND #9 | #5 AND #7 AND #11 |
| #1 AND #14 | #3 AND #8 | #4 AND #10 | #5 AND #12 |
| #1 AND #7 AND #14 | #3 AND #7 AND #8 | #4 AND #7 AND #10 | #5 AND #7 AND #12 |
| #2 AND #6 | #3 AND #9 | #4 AND #11 | #5 AND #13 |
| #2 AND #6 AND #7 | #3 AND #7 AND #9 | #4 AND #7 AND #11 | #5 AND #7 AND #13 |
| #2 AND #8 | #3 AND #10 | #4 AND #12 | #5 AND #14 |
| #2 AND #7 AND #8 | #3 AND #7 AND #10 | #4 AND #7 AND #12 | #5 AND #7 AND #14 |
